# Lewis's Pocket Medical Vocabulary

**Published:** 1891-09

**Authors:** 


*Lewis's Pocket Medical Vocabulary.* Second Edition. Pp. 314. London: H. K. LEWIS. 1891.

We have had only too good reason to mistrust Pocket Vocabularies, which we have usually found almost valueless; but this book is certainly an exception. It is fairly up to date, is well printed, and, within the limits it has set itself, is one that can be recommended.

